# Meaning of Missing Values in Eyewitness Recall and Accident Records

**DOI:** 10.1371/journal.pone.0012539

**Published:** 2010-09-02

**Authors:** Bob Uttl, Kelly Kisinger

**Affiliations:** 1 Mount Royal University, Calgary, Canada; 2 University of Calgary, Calgary, Canada; University College London, United Kingdom

## Abstract

**Background:**

Eyewitness recalls and accident records frequently do not mention the conditions and behaviors of interest to researchers and lead to missing values and to uncertainty about the prevalence of these conditions and behaviors surrounding accidents. Missing values may occur because eyewitnesses report the presence but not the absence of obvious clues/accident features. We examined this possibility.

**Methodology/Principal Findings:**

Participants watched car accident videos and were asked to recall as much information as they could remember about each accident. The results showed that eyewitnesses were far more likely to report the presence of present obvious clues than the absence of absent obvious clues even though they were aware of their absence.

**Conclusions:**

One of the principal mechanisms causing missing values may be eyewitnesses' tendency to not report the absence of obvious features. We discuss the implications of our findings for both retrospective and prospective analyses of accident records, and illustrate the consequences of adopting inappropriate assumptions about the meaning of missing values using the Avaluator Avalanche Accident Prevention Card.

## Introduction

Accident records are frequently used to learn about conditions and participant behaviors before, during, and after accidents. However, accident records frequently do not mention the conditions and/or behavior of interest to researchers and lead to missing values. In turn, missing values lead to uncertainty about the prevalence of conditions and behavior surrounding accidents as researchers are unable to establish from the records whether, for example, a road was dry, wet, or covered by snow. What do these missing values mean? First, accumulated evidence from memory and cognition research demonstrates that if eyewitnesses do not notice conditions or behaviors, they will not remember them and not mention them [1], [2]. Thus, missing values may occur because eyewitnesses did not notice the conditions and behaviors. Second, eyewitness are likely to encode and therefore to remember obvious, distinctive, and important features of events and less likely to remember detailed, non-distinctive, and unimportant features as obvious, distinctive, and important features are typically remembered better [2],[3]. Thus, missing values may occur because the information of interest to researchers was too detailed, non-distinctive, and unimportant for eyewitnesses to encode. Accordingly, we would expect eyewitnesses to encode and to remember such obvious features of accidents as whether it was snowing, raining, or dry and sunny; whether one car hit the other or the other way around; whether visibility was reduced by snow, rain, fog, or darkness; or whether a driver was distracted by looking for a cell phone dropped on a car floor.

However, even though eyewitnesses encode specific information and even though they remember it, they may not necessarily report it when asked to describe what happened. Intuitively, eyewitnesses typically have no reason to report the *absence* of conditions that, when present, increase the risk of accidents (e.g., rain and snow in case of motor vehicle accidents). They are more likely to focus on and report the *presence* of conditions and behavior that they believe cause or contribute to the accident's occurrence (e.g., rain, snow, reduced visibility, distraction, failure to yield). Similarly, people who experience problems with their cars tend to report to their mechanics the presence of problems that indicate something is wrong (e.g., clunking sound from the engine compartment) and are unlikely to give the mechanic a long list of the car components that are working just fine (e.g., the windshield is not broken and does not need to be looked at). Thus, missing values in accident records can occur for at least two main reasons: eyewitnesses did not encode the sought-after information because they did not notice it, and/or eyewitnesses did encode the information (e.g., the absence of obvious clues) but did not report it [4].

When analyzing accident records for the presence or absence of obvious features that any victim or observer would notice, researchers often assume that if obvious features are not mentioned in records, they did not occur (i.e., that missing values indicate absence) [5]–[7]. For example, in a recent narrative text analysis of tractor fatality reports, the researchers assumed that when a report did not mention a tractor rollover, the rollover did not occur [5] and retained all accident records in their analyses. More conservatively, their findings represent the lower bound on the presence of various accident features. Yet another approach to missing values – the list-wise elimination of all records with missing values – was taken by Haegeli and McCammon [8], [9] in developing the Avaluator Avalanche Accident Prevention Card designed to reduce the number of avalanche accidents in Canada. Specifically, Haegeli and McCammon started with over 1,400 avalanche accident records and for each accident record they determined whether each of the seven so-called Obvious Clue (i.e., clues that any participant or eyewitness is certain or nearly certain to notice) was present, absent, or indeterminate from the accident record. Next, they eliminated 1,142 records or 82% of their sample because the status of at least one of the obvious clue was indeterminate (i.e., resulted in a missing value) and used only the remaining 252 records (18% of their original sample) to develop the Avaluator [10], [11]. However, this listwise deletion approach is appropriate if and only if the missing values occurred due to some purely random process [10], [12]–[14]. If the missing values occurred because, for example, accident victims and eyewitnesses are more likely to report the presence than the absence of obvious clues, then the deletion of 82% of the accidents would shift the distribution of the obvious clues towards more clues and result in inflated prevention values.

**Figure 1:**
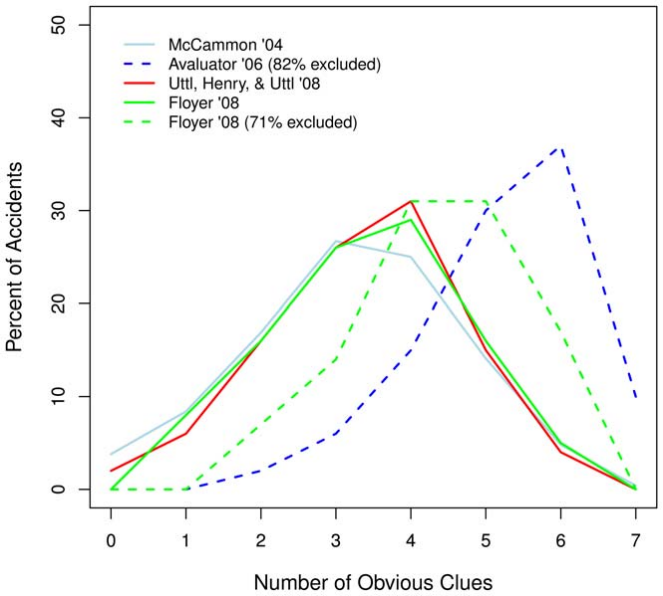
Distributions of Obvious Clues with missing values either included or excluded. Three studies ([11], [7], [15]) which reported the distribution of the obvious clues based on all accidents in their sample found nearly identical distributions of obvious clues. In contrast, the two studies that excluded accidents due to missing values – the Avaluator (82% of accidents excluded; [8]) and Floyer (71% of accidents excluded; [11]) – reported obvious clue distributions that are markedly shifted towards a higher number of clues.

Figure 1 illustrates the problem. Different assumptions about the meaning of missing values taken by researchers analyzing the same and/or nearly the same accident data sets resulted in vastly different distribution of the Obvious Clues. Figure 1 shows the distribution of obvious clues in US avalanche accidents reported when accidents with missing values are either included or excluded. Three studies [11], [7], [15] which reported the distribution of obvious clues based on all accidents in their sample found nearly identical distributions of obvious clues. In contrast, the two studies that excluded accidents due to missing values [11], [8] reported inconsistent results and obvious clue distributions that are markedly shifted towards a higher number of clues. The substantial differences observed between the distributions when accidents with missing values are included vs. excluded strongly suggest that missing values do not occur due to some random process but rather are caused by victims, rescuers, and eyewitnesses not reporting the absence of obvious clues.

Surprisingly, to our knowledge, no prior study has systematically investigated whether eyewitnesses are likely to report the absence of obvious conditions and behaviors of witnessed accidents that increase the probability of accident and that, when present, would be considered obvious clues of increased accident risk normally noticed by all or nearly all observers. In the present study, we used car accidents to examine the probability that eyewitnesses report the presence vs. absence of obvious features of accidents. Participants in the current study were shown video clips of car accidents and subsequently asked to recall as much information as they could remember about the accident. Subsequently, participant recalls were coded for the presence and absence of the obvious clues to accident danger (e.g., snow, rain) and the probabilities of reporting the presence vs. absence of the obvious clues were calculated. To determine if the participants noticed and remembered the status of the obvious clues that they failed to report in their recalls, the participants were also given a multiple choice accident questionnaire asking about various conditions and behaviors observed in the witnessed accidents.

## Methods

The study was approved by the Red Deer College Research Ethics Board and all participants provided written informed consent prior to participation in the study.

### Participants and Design

Participants were 240 college students (mean age  = 22.4 years, range  = 18 to 56 years with 89.5% of participants between 18 and 30 years of age). Fifty-nine were males and 180 were females, and one did not disclose his/her gender. English was the first language of 90.4% of participants.

For primary analyses, the design had one between-subjects factor, the obvious clue condition, with three levels: no clues (i.e., no snow and no rain clue), rain clue (i.e., rain clue and no snow clue), and snow clue (i.e., snow clue and no rain clue). For secondary analyses, three additional obvious clues were considered – reduced visibility, failure to yield, and distraction while driving – for a total of five clues.

### Materials

For primary analyses, eight car accidents were selected from movies: four with no rain and no snow clues (no clues), two with rain clue but no snow clue (rain clue), and two with snow clue but no rain clue (snow clue). The no clue accidents were from *Erin Brockovich* (2000), *Driving Miss Daisy* (1989), *No Country For Old Men* (2007), and *Changing Lanes* (2002). The rain clue accidents were both from *Identity* (2003). The snow clue accidents were from *Misery* (1990) and *The Human Stain* (2003). The shortest clip was 14 s and the longest clip was 59 s long. All movie clips were presented in their original DVD quality with sound turned on.

For secondary analyses, five obvious clues were considered: the snow and rain clues (each present in two accidents) and three additional clues that appeared in at least two of the eight movie clips. The additional clues were: reduced visibility (visibility clue), failure to yield (yield clue), and being distracted while driving (distraction clue). The visibility clue was present in four accidents (*Identity* Clip 1, *Identity* Clip 2, *Misery*, *Human Stain*), the yield clue was present in five accidents (*Identity* Clip 1, *Human Stain*, *Changing Lanes*, *No Country For Old Men*, *Erin Brockovich*), and the distraction clue was present in three accidents (*Identity* Clip 1, *Misery*, *Changing Lanes*). One accident (*Driving Miss Daisy*) had none of the five clues present.

### Procedure

As part of a larger study lasting 1.5 to 2 hours, participants tested in small groups watched a randomly-assigned movie clip and immediately thereafter their memory for accident details was queried using undirected recall, directed recall, and an accident questionnaire, in this order. Prior to watching the movies, participants were told that they would be asked about the accidents later. For undirected recall, participants were instructed “to write down as much as you can remember about what happened in the movie clip. Please be specific and provide as much detail as you can remember.” For directed recall, participants were given the following instructions: “Imagine you were approached by a police officer who wants to reconstruct the accident you observed. Is there anything else you would like to add? Please write it down, be specific, and provide as much detail as you can remember.” The accident questionnaire examined participants' memory for various aspects of accidents including driver, driver conditions, road conditions, and visibility, using checklists. Participants were asked to mark all items on the checklists that applied to the accident they saw. Finally, participants were also asked to indicate whether or not they had seen each movie clip previously.

Accident records (recall protocols) were coded for the presence or absence of these “obvious clues” to accident danger (snow/ice, rain, poor visibility, failure to yield, distraction) using the following scale: Yes  =  the clue was present, Weak Yes  =  the clue was probably present, DNK  =  presence or absence of the clue is unclear from the record, Weak No  =  the clue was probably absent, No  =  the clue was absent [15], [16].

## Results

The recall analyses below are from the first undirected recall. The data from the second, directed recall did not change the pattern of findings because participants rarely provided more complete information about either the absence or presence of the clues. Although some participants reported that they had seen some of the movie clips previously, the exclusion of their data did not alter the findings, and thus, their data were retained in the analyses.

### Primary Analyses

**Figure 2:**
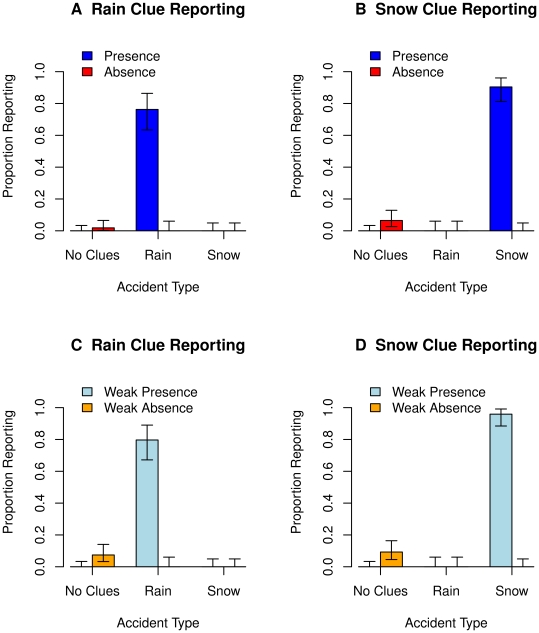
Reporting the presence vs. absence of the rain and snow clues. The proportions of participants reporting the presence vs. absence of the rain (panels A and C) and snow (panels B and D) by the clue condition (No Clues, Rain Clue, Snow Clue) using the strict (Yes and No; panels A and B) and liberal (Yes+Weak Yes and No+Weak No, panels C and D) criteria. Participants were very likely to report the presence of the clues but rarely reported the absence of the clues.

Figure 2 shows the proportion of participants reporting the presence and absence of the rain and snow clues by clue condition (No Clues, Rain Clue, Snow Clue) using the strict (Yes and No) and liberal (Yes+Weak Yes and No+Weak No) criteria with error bars indicating 95% Confidence Intervals. When the clues were present, participants were very likely to report it (strict criteria: *p* = .83, liberal criteria: *p* = .88). In contrast, when the clues were absent, participants rarely reported their absence (strict criteria: *p* = .04, liberal criteria: *p* = .08). No participants erroneously reported the presence of the clues when they were actually absent and similarly no participants erroneously reported the absence of the clues when they were actually present.

**Figure 3:**
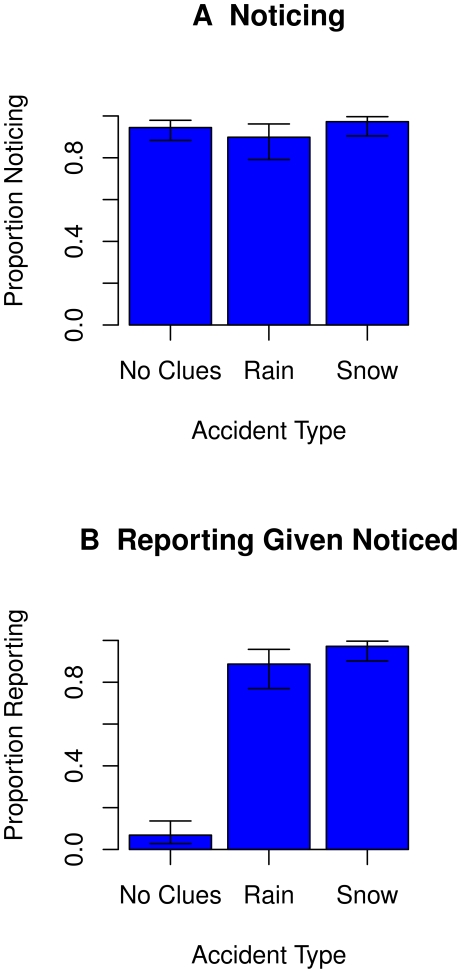
Noticing and reporting clue status. Panel A shows the proportions of participants noticing clue status by the clue condition. Panel B shows the proportions of participants reporting the clue status given that they noticed the status by the clue condition. Participants noticed the absence of the clues but choose to not report it.

Figure 3, panel A, shows the proportion of participants noticing the status of clues – the presence of neither rain nor snow clue (No Clues), the presence of rain clue (Rain) and the presence of snow clue (Snow) – as revealed by their choices on the accident questionnaire. The error bars represent 95% Confidence Intervals. The figure indicates that the vast majority of the participants noticed the actual status of the clues – the absence of rain and snow, the presence of rain, and the presence of snow – in the three accident types (No Clues, Rain, Snow). Thus, participants' failure to report the absence of clues is not due to their failure to notice and/or to encode their absence.

Figure 3, panel B, shows the proportion of participants reporting the absence of rain and snow clues, the presence of the rain clue, and the presence of the snow clue given that they noticed the clue status (i.e., the proportion of participants who reported on the clue status out of those who noticed their status as indicated by their responses on the accident questionnaire). The error bars represent 95% Confidence Intervals. The data highlight that even though the participants knew the clues were absent they did not mention their absence. In contrast, when participants knew the clues were present, they were very likely to mention their presence.

### Secondary Analyses

**Figure 4:**
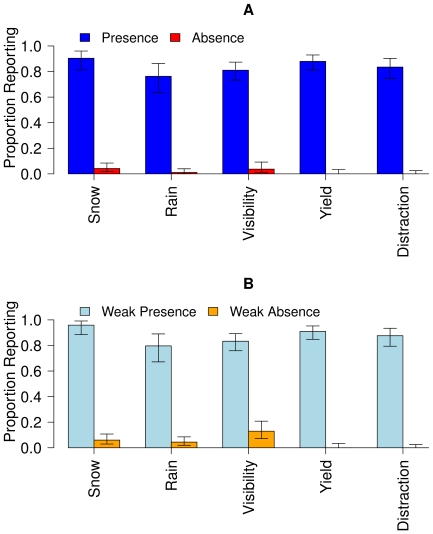
Reporting the presence vs. absence of the five clues. The proportion of participants reporting the presence vs. absence of the five clues (snow, rain, visibility, yield, distraction) using the strict (Yes and No, panel A) and liberal (Yes+Weak Yes and No+Weak No, panel B) criteria. For all five clues, participants were very likely to report the presence of the clues but rarely reported the absence of the clues.

Figure 4 shows the proportion of participants reporting the presence vs. absence of the five clues using the strict (Yes and No) and liberal (Yes+Weak Yes and No+Weak No) criteria with error bars indicating 95% Confidence Intervals. Participants were very likely to report the presence of the obvious clues (strict criteria: *p* = .84, liberal criteria: *p* = .87) and were very unlikely to report their absence (strict criteria: *p* = .02, liberal criteria: *p* = .05).

**Figure 5:**
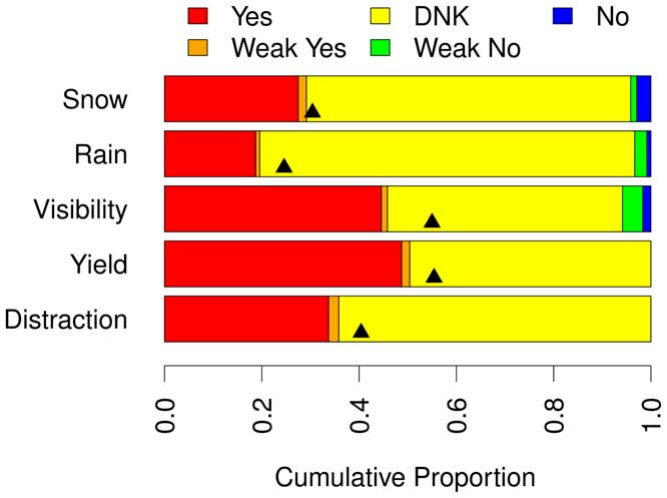
Reported vs. actual status of the five clues. The proportions of accidents falling into each of the five clue status coding categories (Yes/Present, Weak Yes/Probably Present, DNK/Status is indeterminate, Weak No/Probably absent, No/Absent) for the five clues and the true status of the clues (marked by the triangles). The vast majority of missing values (i.e., DNK, yellow portions) occurred because the clues were actually absent.

Figure 5 shows the prevalence of Yes (clue is present), Weak Yes (clue is probably present), Unknown/DNK (presence or absence of clue cannot be established), Weak No (clue is probably absent), and No (clue is absent) judgments for the five clues. The DNK portion highlights that accident reports themselves do not allow us to determine the presence or absence of obvious clues for large portions of accidents. However, the upwards pointing black triangles in the figure show the true prevalence of the obvious clues in the movie clips shown to the participants. Consistent with the analyses above, the vast majority of missing values (DNK, yellow portion) in analyses of the accident records occurred because the clue was actually absent.

## Discussion

This study revealed several important findings. First, eyewitnesses reported the presence of present obvious clues and only rarely reported the absence of absent obvious clues. In turn, the accident records themselves do not allow researchers to determine whether clues were present or absent in the vast majority of cases, resulting in many missing values. Second, the multiple choice test results revealed that eyewitnesses were fully aware that the obvious clues to accident danger were absent but failed to report their absence. Thus, failure to report the absence of absent clues is not due to eyewitnesses not noticing their absence. Rather, one of the principal mechanisms causing missing values is eyewitnesses' tendency not to talk about the absence of obvious clues. Third, the vast majority of missing values occurred because the obvious clues were actually absent. In turn, the estimated prevalence of the obvious clues under the assumption that the missing values mean the absence of the clues was very close to the actual prevalence for all of the obvious clues.

The current results are consistent with the findings of Uttl, Henry, and Uttl [15]. Uttl et al. analyzed avalanche accident records for the presence or absence of the Obvious Clues using the Avaluator. They found that the status of the Obvious Clues could not be determined in the vast majority of accidents because victims, rescuers, and eyewitnesses did not mention anything about their presence or absence. More importantly, using external objective weather records and avalanche bulletin data issued at the time of accidents, they found that for at least the two clues – Unstable Snow and Thaw – missing values meant that the clues were actually absent. In combination, these findings indicate that the listwise deletion of 82% of accident records by Haegeli and McCammon [8], [9] in the development of the Avaluator was inappropriate as the missing values were caused by eyewitnesses and rescuers not reporting the absence of absent clues.

Our study is the first one to systematically examine whether eyewitnesses are more likely to report the presence vs. absence of obvious features and behaviors and is limited by the use of car accidents only. However, as noted above, our results are consistent with the conclusions reached by Uttl et al. [15] for real-life avalanche accident records based on eyewitness accounts of victims, bystanders, and rescuers rather than laboratory findings with college students. They are also consistent with the findings by Lindsay et al. [4] for eyewitness descriptions of perpetrators of crime. Thus, we expect our results to generalize to other populations, other accident types, as well as other eyewitness situations.

Importantly, as discussed in the introduction, these findings are applicable to only obvious features of accidents and behaviors, that is, those features that any victim, observer, or investigator is sure to notice and consider relevant. For example, a skier working his or her way for hours through 50 cm of fresh snow is nearly certain to encode the presence of fresh snow (so-called Snow Loading clue of the Avaluator's Obvious Clues Method) and likely to report its presence due to its high relevance to avalanche accidents and their outcomes. These findings are not applicable to the interpretation of missing values that may have occurred because victims, eyewitnesses, or investigators did not notice the specific features or behavior.

Our findings have widespread implications for theories and models of eyewitness memory as well as accident victims' behavior. Although the model of tractor fatalities may be valid [5], the Avaluator's behavioral recommendations based on the Obvious Clues distribution in historical avalanche accidents are likely invalid and dangerous [15], [16], [10]. List-wise deletion of 82% of data due to missing values (which is also, incidentally, the default behavior of some of the most widely-used statistical programs such as *SPSS*) will rarely result in unbiased statistics and certainly not if eyewitnesses are far more likely to report the presence than the absence of obvious clues as suggested by the present study.

These findings have different implications for conducting retrospective analyses of historical records vs. prospective analyses of future accidents and events (including experimental studies of memory). Historical records are limited to the information gathered by record keepers at the time and researchers are unable to go back in time and inquire about the features and behaviors of interest. When missing values occur because the records do not contain the sought-after information, researchers should first attempt to establish the meaning of missing values from external data whenever possible (e.g., from historical weather data, see Uttl et al. [15]). Alternatively, if the meaning of missing values cannot be established, researchers may impute missing data using clearly specified assumptions about the meaning of missing values. Unfortunately, if the chosen assumptions are wrong, so too will be the researchers' interpretations of their findings.

To illustrate, in the development of the Avaluator, Haegeli and McCammon [8], [17] assumed, without stating so, that missing values occurred due to some purely random process (i.e., in technical terms, were missing completely at random, [13], [14], [18]), that is, that the 82% of accidents they excluded from their analyses were no different from the 18% of accidents that they retained. Based on the remaining accident records, Haegeli and McCammon [8] concluded that 77% of accidents occurred when 5, 6 or 7 of the Obvious Clues were present (see Figure 1), and therefore, if the historical victims had avoided slopes with 5 or more of the Obvious Clues, 77% of the accidents would not have occurred (i.e., the use of the Avaluator's behavioral recommendation would result in a 77% relative risk reduction for the victims being involved in avalanche accidents). In turn, every student in Avalanche Safety Training courses in Canada approved by Canadian Avalanche Association has been given the Avaluator [19] and taught that slopes with 0 to 4 clues are relatively safe whereas slopes with 5 or more clues are dangerous and should be avoided [8], [20]. However, as shown by the present study as well as Uttl et al's [15] findings using historical weather data, Haegeli and McCammon's [8] implicit assumption is incorrect and, in this instance, the missing values arose primarily because victims, eyewitnesses, and rescuers did not report the absence of obvious clues. Accordingly, if accidents with missing data are not excluded and missing values are imputed with zeros (i.e., absence of Obvious Clues), the obtained distribution of the Obvious Clues is shifted towards a much lower number of clues (see Figure 1) and the behavioral recommendations are vastly different. To avoid approximately 80% of historical accidents (i.e., achieve 80% relative risk reduction), victims would have to avoid slopes with 3 or more clues and only slopes with 0, 1, or 2 clues could be considered relatively safe (see Figure 1).

In contrast, for prospective analyses of future accidents and events, researchers are best advised to use structured interviews or multiple choice questionnaires that directly ask victims, eyewitness, and investigators whether certain features or behaviors of interest were present or absent. Indeed this is the approach increasingly taken in investigations of motor vehicle accidents where investigators are often required to complete multiple choice questionnaires asking about every conceivable piece of information that may be of interest in the future.

More generally, our study underscores the importance of reporting the extent of missing values as well as patterns of their distribution, to consider why missing values occurred and their meaning, and to consider the impact of their treatment (e.g., deletion, replacement with zeros) on statistical description and inference [18], [12], [14], [21], [22]. A failure to do so may lead not only to unreplicable findings but also to dangerous public policies based on the esoteric properties of small and unrepresentative accident samples resulting from data exclusion due to missing values [10], [15], [16], [23], [24].
